# Expression, Function and Trafficking of the Human ABCG2 Multidrug Transporter Containing Mutations in an Unstructured Cytoplasmic Loop

**DOI:** 10.3390/membranes13100822

**Published:** 2023-10-04

**Authors:** Orsolya Mózner, Boglárka Zámbó, Zsuzsa Bartos, Anna Gergely, Kata Sára Szabó, Bálint Jezsó, Ágnes Telbisz, György Várady, László Homolya, Tamás Hegedűs, Balázs Sarkadi

**Affiliations:** 1 Institute of Enzymology, Research Centre for Natural Sciences, 1117 Budapest, Hungary; mozner.orsolya@ttk.hu (O.M.); homolya.laszlo@ttk.hu (L.H.); 2 Doctoral School, Semmelweis University, 1085 Budapest, Hungary; 3 Department of Biochemistry, Eötvös Loránd University, 1117 Budapest, Hungary; 4 Department of Biophysics and Radiation Biology, Semmelweis University, 1094 Budapest, Hungary; 5 TKI-SE Biophysical Virology Research Group, 1094 Budapest, Hungary

**Keywords:** ABCG2, BCRP, MXR, multidrug transporter, unstructured loop variants

## Abstract

The human ABCG2 multidrug transporter plays a crucial role in the absorption and excretion of xeno- and endobiotics, contributes to cancer drug resistance and the development of gout. In this work, we have analyzed the effects of selected variants, residing in a structurally unresolved cytoplasmic region (a.a. 354–367) of ABCG2 on the function and trafficking of this protein. A cluster of four lysines (K357–360) and the phosphorylation of a threonine (T362) residue in this region have been previously suggested to significantly affect the cellular fate of ABCG2. Here, we report that the naturally occurring K360del variant in human cells increased ABCG2 plasma membrane expression and accelerated cellular trafficking. The variable alanine replacements of the neighboring lysines had no significant effect on transport function, and the apical localization of ABCG2 in polarized cells has not been altered by any of these mutations. Moreover, in contrast to previous reports, we found that the phosphorylation-incompetent T362A, or the phosphorylation-mimicking T362E variants in this loop had no measurable effects on the function or expression of ABCG2. Molecular dynamics simulations indicated an increased mobility of the mutant variants with no major effects on the core structure of the protein. These results may help to decipher the potential role of this unstructured region within this transporter.

## 1. Introduction

The human ABCG2 protein functions as a homodimeric plasma membrane multidrug transporter and plays an important role in the ADME-Tox properties of a large number of pharmacological agents. This ATP-dependent exporter is also involved in the cellular extrusion of endobiotics, including uric acid, thus preventing the development of gout [1,2,3]. In addition, ABCG2 overexpression in various cancers causes anticancer drug resistance [4,5]. Therefore, the regulation of the expression and cellular trafficking of ABCG2 modulates numerous medical conditions and/or treatment potentials. Naturally occurring mutations in this transporter have been shown to significantly alter expression levels and plasma membrane appearance, as well as the transport capacity of ABCG2 [6,7,8,9].

**Figure 1:**
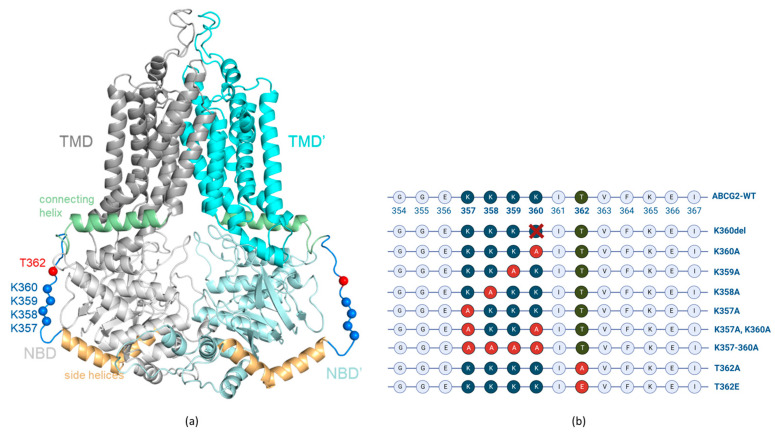
(**a**) ABCG2 (PDBID: 5hij) structure with modelled loops. Gray, cyan: TMDs; light gray, pale cyan: NBDs; orange: side helices (a.a. 328–353); pale green: connecting helices (a.a. 368–391); blue: disordered loop (a.a. 354–367); blue spheres: Cα of lysine residues from 357 to 360; red spheres: Cα of T362. (**b**) Schematic representation of the localization of the engineered ABCG2 variants in the unstructured loop (aa. 354–367) region.

Several atomic-level structures of the active homodimer form of this protein are available by now, with or without bound ATP and/or transported substrate molecules [10,11,12]. However, there are some highly flexible unstructured cytoplasmic regions of ABCG2 which are not resolved in any of the published structures. In the present work, we have depicted one of these structurally unresolved cytoplasmic regions, a loop of amino acids from 354 to 367 (see Figure 1), and analyzed the effects of selected variants within this loop.

The loop region in ABCG2 contains four neighboring lysines (K357–360). Similar motifs, i.e., clusters of three or four lysines, have been described in several membrane proteins and been shown to significantly affect their organellar distribution and/or polarized trafficking [13,14,15]. There is a naturally occurring K360del variant in the human population (rs750972998) [16]; in our previous studies and those of Toyoda et al. [17,18], only minor differences in the localization and function of this variant were observed.

The unstructured cytoplasmic loop of amino acids from 354 up to 367 also contains a potentially phosphorylated threonine (T362) residue, which was reported to be selectively phosphorylated by PIM kinase [19]. Moreover, this PIM kinase-dependent T362 phosphorylation was suggested to be crucially important for both the membrane insertion and function of the ABCG2 transporter [19]. It has also been suggested that the inhibition of this kinase and the connected PI3K/Akt signaling pathway, activated in many human tumors, might be an important drug target for reducing ABCG2-related cancer drug resistance [20]. 

In order to explore the role of the lysine cluster, we have generated K357A, K358A, K359A and K360A single mutants, the K357A/K360A double mutant, as well as a four-alanine mutant, K357A-K358A-K359A-K360A (referred to as K357–360A), ABCG2 variants (Figure 1b), and analyzed the function, trafficking and polarized localization of these variants. For studying the potential effect of the T362 phosphorylation state, we have generated the T362A and T362E ABCG2 variants, mimicking either the non-phosphorylated or the negatively charged, phosphorylated residue. Again, we measured both the function and the localization of these transporter variants. In these experiments, we applied mammalian cell expression systems (HEK, HeLa and MDCKII cells) in which the expression of the untagged or the GFP-tagged ABCG2 variants could be achieved and the glycosylation, plasma membrane insertion, transport function and the cellular trafficking could all be followed. In this latter regard, we have applied the dynamic RUSH system [21], as described in detail by Bartos and Homolya, 2021 [22], to determine the velocity of the surface delivery of the ABCG2 variants. In addition, their internalization was assessed by following the loss of the membrane-inserted transporter protein by inhibiting cellular protein synthesis. 

In addition to these experimental studies, we have performed molecular dynamics simulations for both the wild-type and the loop mutant variants of the full length ABCG2 as earlier studies suggested a potential role of this cytoplasmic unstructured region in communicating allosteric signals [23]. The results obtained here should clarify some of the questions related to earlier results and help to decipher the potential role of this unstructured region within the ABCG2 transporter.

## 2. Materials and Methods

### 2.1. Vector Constructs

The p10-ABCG2-IRES2-GFP coding vector was used in all experiments that involved the expression of untagged ABCG2 variants. Mutations were introduced in the ABCG2 coding sequence by mutagenesis PCR and cassette change. All vector sequences were checked by Sanger sequencing. The p10-ABCG2-IRES2-GFP vector is a transposon coding vector. For stable cell line generation by the transposon–transposase method, it was used together with the SB100 Sleeping Beauty transposase coding vector in 9:1 molar ratio (p10:SB100 vector) [24]. For transient expression, it was used without the transposase vector.

For the dynamic trafficking experiments, RUSH vectors containing N terminally tagged ABCG2 variants and an endoplasmic reticulum (ER) hook were used as described in Bartos and Homolya [22]. Mutations were introduced into these plasmids from the above-mentioned templates, by replacing the WT-ABCG2 coding sequence with the examined ABCG2 mutant coding sequences. The mutant ABCG2 coding new plasmids were checked by Sanger sequencing. 

### 2.2. Cell Culture and Transfection

The HEK293H (Gibco, Thermo Fisher Scientific, Waltham, MA, USA, cat. CVCL_6643), HeLa (ATCC, Manassas VA, USA, cat. CCL-2) and MDCKII (ECACC 00062107) cell lines were used in the experiments. Cells were grown in DMEM/high glucose/GlutaMAX medium (Gibco, Thermo Fisher Scientific, Waltham, MA, USA, cat. 10569010) completed with 10% FBS (Gibco, Thermo Fisher Scientific, Waltham, MA, USA, cat. 1640071) and 1% Penicillin-Streptomycin (Gibco, Thermo Fisher Scientific, Waltham, MA, USA, cat. 15070063) at 37 °C (5% CO_2_). Transfection of HEK293 and HeLa cells was carried out with Lipofectamine 2000 (Invitrogen, Thermo Fisher Scientific, Waltham, MA, USA, cat. 11668019) in Opti-MEM medium (Gibco, cat. 31985070), according to the manufacturer’s protocol. Protein determination and Hoechst dye extrusion measurements were carried out 48 h after transfection.

For cycloheximide treatment, 20 µg/mL final CHX concentration in completed DMEM/high glucose/GlutaMAX was used. Normal growth medium was replaced for CHX medium for 1 or 4 h. We used a 100 mg/mL CHX in DMSO stock solution (Sigma Aldrich, Burlington, MA, USA, cat. C4859).

### 2.3. Generation of Stable Cell Lines

The ABCG2-expressing stable cell lines were generated using the Sleeping Beauty transposon–transposase system. MDCKII cells were transfected with the mixed plasmids using Lipofectamine 2000 (Invitrogen, Thermo Fisher Scientific, Waltham, MA, USA, cat. 11668019) in Opti-MEM (Gibco, Thermo Fisher Scientific, Waltham, MA, USA, cat. 31985070). Four days after transfection, the GFP positive cells were sorted using BD FACS Aria III and further cultured. The result was a mixed culture where several cells were stably expressing the ABCG2 variant; this was suitable for our future analysis to examine the localization of ABCG2 in the polarized MDCKII cells.

### 2.4. Hoechst33342 Uptake Measurements

To examine the transport function of ABCG2 in transfected HEK293 and HeLa cells, 3 days after transfection with the ABCG2-IRES2-GFP coding plasmids, trypsinized cells were incubated and gently shaken for 20 min at 37 °C in HPMI buffer (20 mM HEPES, 132 mM NaCl, 3.5 mM KCl, 0.5 mM MgCl_2_, 5 mM glucose, 1 mMCaCl_2_, pH 7.4) with 1 µM Hoechst33342 (Thermo Fisher Scientific, Waltham, MA, USA, cat. H1399) after preincubation for 5 min with the presence or absence of 1 µM of the Ko143 (Tocris Bioscience, Bristol, UK, cat. 3241) ABCG2 inhibitor. Following incubation with the dye, cells were put on ice until measurement. Hoechst33342 fluorescence of the cells was measured by the Attune NxT Cytometer (Thermo Fisher Scientific, Waltham, MA, USA). GFP fluorescence was detected in the BL1, while Hoechst33342 fluorescence was detected in the VL1 detector. For data analysis, the Attune NxT Cytometer Software v3.1.2 was used. MAF (Multidrug resistance activity factor) values [25] were calculated from the results the following way: MAF = (F_(inh)_ − F_(no inh)_)/F_(inh)_, where F_(inh)_ is the median of Hoechst fluorescence of the cells with the inhibitor, while F_(no inh)_ is the fluorescence of the cells without the inhibitor. When comparing these results to cell surface expression levels, the activity factor was divided by cell surface expression levels, measured by flow cytometry after ABCG2-specific 5D3 antibody labeling. 

### 2.5. Plasma Membrane Expression Measurement

Cell surface expression of ABCG2 was determined in the transfected HEK293 and HeLa cells 48 h after transfection. Trypsinized cells were incubated in small volumes (100 µL) and gently shaken for 40 min at 37 °C in 0.5% BSA/PBS with 1 µM Ko143 (Tocris Bioscience, Bristol, UK, cat. 3241) and the ABCG2-specific 5D3 mouse monoclonal antibody (gift of Bryan Sorrentino, Division of Experimental Hematology, Department of Hematology/Oncology, St. Jude Children’s Research Hospital). Ko143 was added to the samples because it has been shown to help the conformation-sensitive 5D3 antibody recognition. Cells were washed twice with 2 mL PBS. After washing, the cells were centrifuged and the supernatant discarded, then incubated for 30 min at 37 °C with the secondary antibody (goat anti-mouse IgG2b Alexa Fluor 647, cat. A-21242, Invitrogen, Thermo Fisher Scientific, Waltham, MA, USA) in 0.5% BSA/PBS. Cells were washed again with 2 mL PBS. Measurements were performed in the Attune NxT Cytometer (Thermo Fisher Scientific, Waltham, MA, USA) using the RL1 detector. For data analysis, the Attune NxT Cytometer Software v3.1.2 was used.

### 2.6. Western Blotting

Total protein from the cells was extracted by the addition of TE sample buffer (0.1 M TRIS-PO_4_, 4% SDS, 4 mM Na-EDTA, 40% glycerol, 0.04% bromophenol blue, and 0.04% β-mercaptoethanol; materials from Sigma-Aldrich, Burlington, MA, USA). Equal amounts of the protein samples were loaded on 10% acrylamide gels. Blots were probed with the following primary antibodies: anti-ABCG2 (BXP-21, Abcam, Cambridge, UK, cat. ab3380) and anti-GFP (Abcam, Cambridge, UK, cat. ab290). Goat anti-mouse IgG (H+L) HRP conjugate (Abcam, cat. ab97023) and goat anti-rabbit IgG (H+L) HRP conjugate (Abcam, Cambridge, UK, cat. ab6721) secondary antibodies were used to visualize and quantify the results. Detection was performed with Clarity Western ECL Substrate (BioRad, Hercules, CA, USA, cat. 1705060) and luminescence was detected with the BioRad ChemiDoc MP Imaging System. Densitometry analysis was performed by ImageJ software v1.42q.

### 2.7. Immunostaining and Confocal Imaging of MDCKII Cells

MDCKII cells were cultured under polarizing conditions on Corning Costar 24-well transwell plates with 24-well transwell insert polyester membranes (Corning Inc., Corning, NY, USA, cat. 3470) for 5 days. The membrane was cut from the transwell and transferred to microscope slides after immunostaining. For immunostaining, cells on the transwell were gently washed with PBS and fixed with 4% paraformaldehyde in PBS for 5 min at room temperature, followed by permeabilization in methanol for 5 min on ice.

After washing, samples were blocked for 1h at room temperature with 2% BSA, 1% fish gelatin, 0.1% Triton-X 100, 5% goat serum in PBS (blocking buffer), and all antibodies were diluted in blocking buffer in the following steps. Samples were incubated for 1 h at room temperature with primary ABCG2 antibody (mouse, Bxp-21 1:200, Abcam, Cambridge, UK, cat. Ab3380) and Na,K-ATPase antibody (chicken, 1:500, Abcam, Cambridge, UK, ab353), which was used as a basolateral marker. After washing with PBS, cells were incubated for 1 h at room temperature with the secondary antibodies (1:250, Alexa Fluor 647-conjugated goat anti-chicken, Thermo Fisher Scientific, Waltham, MA, USA, cat. A-21449 and Alexa Fluor 568 goat anti-mouse Thermo Fisher Scientific, Waltham, MA, USA, cat. A-11004). Immunostaining was performed in the transwell, and for the confocal microscopy, the membrane was cut from the insert and transferred to microscope slides. Cells were imaged by the Zeiss LSM 710 confocal laser scanning microscope, and images were processed with ZEN 2012 software, blue edition.

### 2.8. Confocal Microscopy Imaging and Kinetic Analysis

To analyze the cellular trafficking of the ABCG2 variants in detail, we employed the dynamic RUSH system [21], as previously developed by Bartos and Homolya, 2021 [22]. Briefly, the ABCG2 variant tagged with streptavidin-binding peptide (SBP) and GFP, as well as an ER-resident hook protein, i.e., the invariant chain (Ii) of major histocompatibility complex (MHC) class II tagged with streptavidin, were transiently co-expressed in HeLa cells. In these cells, the ABCG2 variant was retained in the ER. To release ABCG2 from the ER and to monitor its cell surface expression, 24 h after transfection the cells were subjected to 100 µM biotin and Alexa Fluor 647 conjugated 5D3 antibody (1 µg/mL, Novus Biologicals, Littleton, CO, USA, cat. FAB995R) plus 1 µM Ko143 (Sigma-Aldrich, Burlington, MA, USA, cat. K2144), respectively. After 1, 2 or 4 h incubation, the samples were gently washed with PBS, fixed with 1% PFA for 5 min at room temperature and washed again three times with PBS. For time point zero, biotin was omitted from the medium. The cells then were imaged by confocal microscopy as specified above. For each condition, six fields of view containing 15–20 transfected cells were acquired in 3–4 biological parallels. The three variants of the ABCG2 examined here were the GFP tagged WT, K360del and the K360A proteins.

For kinetic analysis, the localization-based colocalization coefficients (CC) of red versus green fluorescence (5D3 labeling versus GFP fluorescence, respectively) were derived from the images at each condition using the ZEN 2012 software. The time courses were then fitted with a sigmoidal function using the least square method as described in Bartos and Homolya, 2021 [22]. The kinetic parameters, such as the initial values (CC_0_), the limits of the function (CC_inf_) and the time constants (k), were determined. For statistical analyses, Student’s *t* test was used. Differences were considered significant when *p* < 0.05.

### 2.9. Molecular Dynamics Simulations

The wild type (PDBID: 6hij), K360del and K360A ABCG2 structures with loops and mutations generated by Modeller [26] were used in molecular dynamics simulations. One model based on DOPE score was selected, oriented according to the Orientations of Proteins in Membranes database [27] and inserted in a mixed membrane bilayer using CHARMM-GUI [28,29]. The outer leaflet contained approx. 35:25:25:15% of cholesterol/POPC/PLPC/SSM and the inner leaflet 35:18:17:17:8:5% of cholesterol/POPC/PLPC/POPE/POPS/DMPI25 (1-Palmitoyl-2-oleoylphosphatidylcholine; 1-Palmitoyl-2-linoleoylphosphatidylcholine; 1-Palmitoyl-2-oleoylphosphatidylethanolamine; 1-palmitoyl-2-oleoyl-sn-glycero-3-phosphoserine; Dimyristoyl-inositol-4,5-bisphosphate). KCl was used at 150 mM. Grid for PME (Particle-Mesh Ewald) electrostatics was generated automatically. The number of particles, pressure of 1 bar, and temperature of 310 K were constant. GROMACS 2020 with the CHARMM36m force field was used to run molecular dynamics simulations [30,31]. Each system was energy minimized using the steepest descent integrator, which stopped when the largest force in the system became less than 500 kJ/mol/nm. Three simulations for each construct were forked using the energy minimized system with different velocities to increase sampling. Equilibration was performed in six steps with default CHARMM-GUI parameter files and production runs were run for 1 μs. Root mean square deviation (RMSD) of conformations from the initial structure indicated stable simulations (Figure S2). We extracted and merged the last part of the simulations (0.75–1 μs) for each construct and used these conformations for further analysis. RMSD and root mean square fluctuation (RMSF) were calculated using GROMACS tools. Contact maps were generated by calculating the pairwise Cα distances for each residue with a cutoff value of 7.5 Å using the MDAnalysis package [32]. Molecular visualization was performed using PyMOL (Schrödinger Inc., New York, NY, USA). Graphs were generated using Python’s matplotlib library [33]. 

### 2.10. Statistical Analysis

All experiments were performed with at least two technical and two biological replicates. Error bars show the SEM calculated from the SDs of the measurements. GraphPad Prism 8.0.2 was used for statistical analysis and visualization of the results. ABCG2 cell surface expression levels (5D3), Hoechst33342 dye accumulation results and Western blot quantification results were analyzed by one-way ANOVA, and Dunnett’s multiple comparisons test (95% confidence interval) was performed to compare results to the WT-ABCG2 results. Columns marked with a star showed significant difference (adjusted *p* value < 0.05), compared to the WT-ABCG2 results.

## 3. Results

### 3.1. Expression, Membrane Localization and Function of the K357-K360 and T362 ABCG2 Mutant Variants

In the first set of experiments, we have measured the general expression levels and the cell surface appearances of the K357A, K358A, K359A, K360A, K357-358-359-360A, K360del, T362A and the T362E ABCG2 variants in cell lines stably expressing these proteins. HEK293 and HeLa cells were transfected with the p10-CAG-ABCG2-IRES2-GFP vector coding the examined variants. Expression levels were measured three days after transfection. This IRES-based expression system, the GFP expressed from the same vector as the ABCG2 protein, allowed the evaluation of transfection efficiency based on GFP expression. 

**Figure 2:**
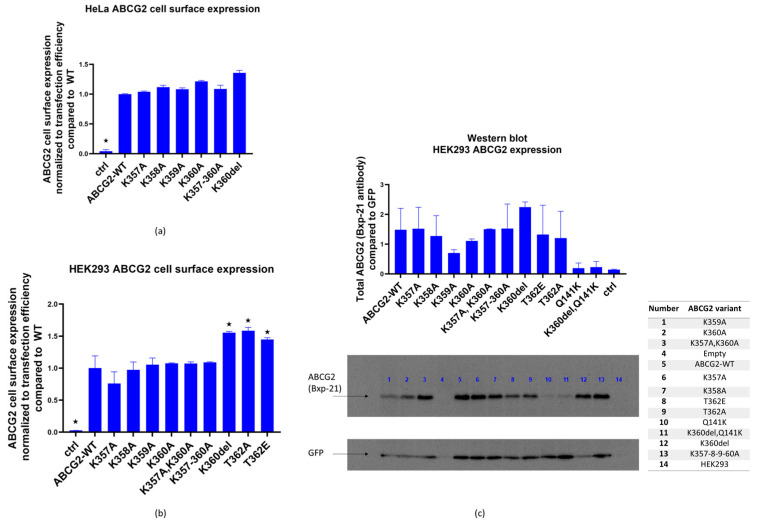
(**a**). ABCG2 cell surface expression (5D3 binding measured by flow cytometry, normalized to GFP fluorescence for transfection efficiency) in HeLa cells 48 h after transfection with plasmids coding the examined ABCG2 variants (WT, K357A, K358A, K360A, K357-8-9-60A, K360del), a plasmid without ABCG2 coding sequence was used as control. (**b**) ABCG2 cell surface expression (5D3 binding measured by flow cytometry, normalized to GFP fluorescence, reflecting transfection efficiency) in HEK293 cells 48 h after transfection with plasmids coding the examined ABCG2 variants (WT, K357A, K358A, K360A, K357-8-9-60A, K360del, T362E, T362A). A similar plasmid without ABCG2 coding sequence was used as control. (**c**) Western blot (Bxp-21 and anti-GFP antibodies) showing ABCG2 expression in HEK293 cells 48 h after transfection. Cells were expressing the examined ABCG2 variants: WT, K357A, K358A, K360A, K357A-K360A, K357-8-9-60A, K360del, T362E, T362A, Q141K and K360del-Q141K, and transfection with a plasmid without ABCG2 coding sequence was used as control. Columns marked with a star showed significant difference (adjusted *p* value < 0.05), compared to the WT-ABCG2 results. See Methods for Statistical Analysis.

As shown in Figure 2a, in most HeLa cells expressing the flexible loop variants, we found similar cell surface expression levels from these ABCG2 variants. We have also estimated the cell surface expression levels of the ABCG2 variants by measuring 5D3 monoclonal antibody binding, selectively interacting with the extracellular portion of the ABCG2 protein [34]. 

As shown in Figure 2a,b, all the ABCG2 variants studied reached the cell surface. The ABCG2 expression was normalized to GFP expression, correcting for potential differences in transfection efficiency and other factors affecting vector-based protein expression. 

In order to study overall cellular expression levels, Western blot measurements were performed 48 h after the transfection of HEK293 cells, where ABCG2 and GFP proteins were detected in total protein samples from the transfected cells (Figure 2c). Again, ABCG2 expression levels were corrected by GFP expression levels. We found variable total expression levels for the variants, with reduced expressions in the cases of K359A and K360A, and an increased level in K360del (these changes, due to the large scattering of the data, were not statistically significant). 

To explore, if the K360del variant may rescue the Q141K-ABCG2 variant, which was shown in several studies to have a lower cellular expression (see [34]), we have also included the Q141K-ABCG2 and the Q141K-K360del double variant in the Western blot measurements generated in this work. As shown in Figure 2c, these variants showed relatively low levels of total cellular expression and the expression level of the double mutant was similar to that of the Q141K mutant. As a consequence, lower cell surface expression levels were seen for both the Q141K and Q141K-K360del mutants. Thus, the K360del mutation did not correct the ABCG2-Q141K expression level.

**Figure 3:**
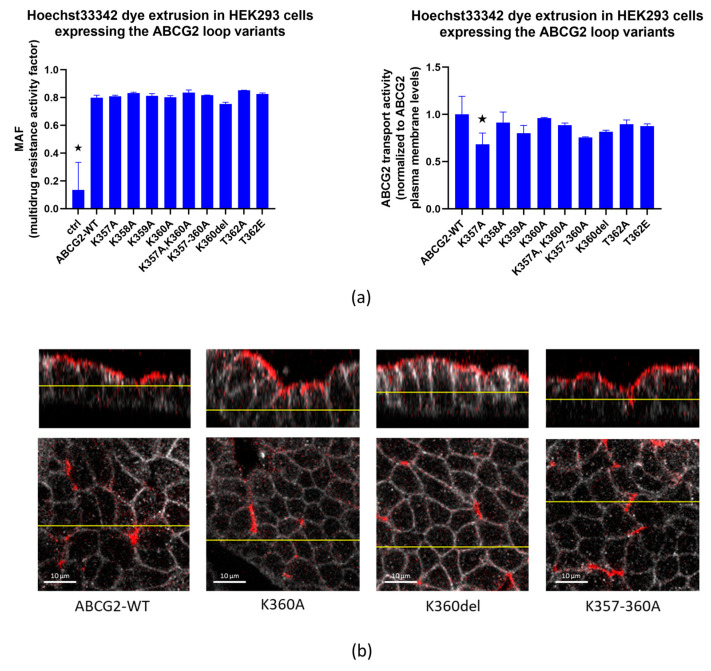
(**a**). Hoechst33342 dye extrusion in HEK293 cells expressing the ABCG2 loop variants. Left Panel: MAF (multidrug resistance activity factor) calculated from flow cytometry measurements of dye uptake with or without the Ko143 inhibitor. Right Panel: transport activity normalized for cell surface expression levels of the ABCG2 variants by 5D3 monoclonal antibody binding levels. (**b**) Confocal images of polarized MDCKII cells expressing ABCG2-WT, K360A, K360del or K357-8-9-60-A variants; red: ABCG2 (Bxp-21 primary antibody, ab3380), white: basolateral Na,K-ATPase (Na,K-ATPase primary antibody, ab353). Columns marked with a star showed significant difference (adjusted *p* value < 0.05), compared to the WT-ABCG2 results. See Methods for Statistical Analysis.

In the following experiments, we measured the transport capacity of the ABCG2 protein in the HEK293 cells transfected by the flexible loop variants. We found that all the ABCG2 variants produced an efficient reduction in the Hoechst33344 dye uptake, representing the dye extrusion activity of the transporter. This transport activity in all cases was eliminated by the specific ABCG2 transporter inhibitor Ko143 (see Figure S1). When the multidrug activity factor (MAF) values were calculated from these experiments (see Methods), these specific activities were similar in the case of all loop variants studied (Figure 3a left panel). When comparing transport activity to cell membrane expression levels (5D3 antibody, flow cytometry results, Figure 3a right panel), a slight decrease in transport function was observed in the case of the K357A variant. 

Data in the literature indicate that the clusters of three or four lysines in unstructured regions of proteins may have significant effects on the cellular distribution, and especially the selective localization of the membrane proteins in polarized cells [35]. Since ABCG2 is an apically localized protein in polarized cells, in the following experiments we examined whether deletion or replacement of the lysines in the flexible loop of this protein alters its polarized localization. Therefore, we have expressed the wild-type protein and the K360del, K360A and K357-360A ABCG2 variants in MDCKII cells cultured under polarizing conditions. To perform these experiments, MDCKII cells were transfected via the p10-CAG-ABCG2-IRES2-GFP coding transposon-based vectors together with the SB100 Sleeping Beauty transposase. This way we created a cell population with the transposon sequence integrated in their genome. These stable cells expressed ABCG2 even weeks after transfection, thus enabling us to examine ABCG2 after cell polarization.

As shown in Figure 3b, all these variants were found to be localized in the apical membrane compartments in these polarized cells. Thus, this lysine cluster in the ABCG2 protein does not alter the proper polarized location of this protein. 

As a summary, these results indicate that all the variants studied here can reach the cell surface and are fully active transporters. While this was expected by earlier results for the K360del variant [17,18], the total replacement of the lysine cluster by alanines still showed proper membrane expression, transport activity and apical membrane localization of ABCG2. The data obtained in these experiments also clearly showed that the removal of the T362 phosphorylation site by replacing it with alanine or glutamic acid did not significantly alter the expression, cell surface appearance or the transport function of this protein.

### 3.2. Effects of Inhibition of Protein Synthesis and Proteasomal Degradation on the Expression of the ABCG2 Loop Mutant Variants

**Figure 4:**
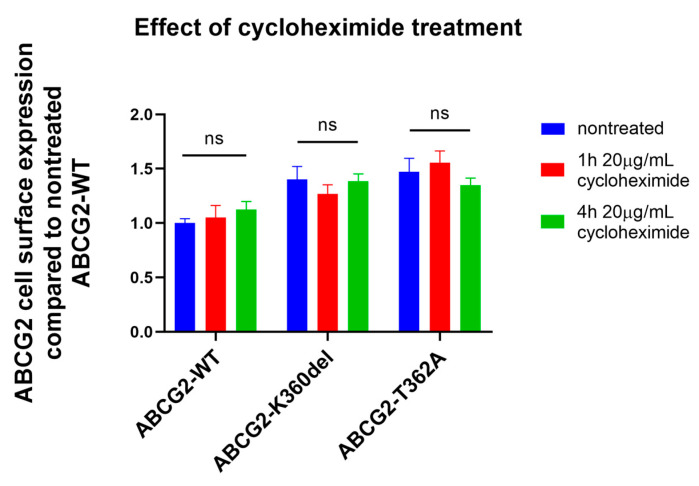
Effect of cycloheximide treatment (1 or 4 h, 20 µg/mL CHX) on ABCG2 cell surface presence (5D3 antibody labeling, measured by flow cytometry) in HEK293 cells transfected with ABCG2-WT, K360del and T362A coding plasmids. CHX treatment was performed 48 h after transfection. For Statistical Analysis, Sutdent’s *t*-test was used. Differences were considered significant when *p* < 0.05, ns: non-significant.

To monitor the internalization of the ABCG2 constructs, in the following experiments we have inhibited cellular protein synthesis by adding cycloheximide (CHX) to the cell culturing media of HEK293 cells, expressing the flexible loop mutant variants. As shown in Figure 4, 48 h after transfection, 1–4 h of CHX treatment did not significantly influence the cell surface expression of any of the ABCG2 variants. It has been shown earlier that wild-type ABCG2 has a long (>60 h) plasma membrane half life [36]; thus, CHX treatment was not expected to decrease the membrane expression levels, while after a longer period this treatment caused significant cell damage and death. Interestingly, internalization of neither the K360del nor the T362A variant was significantly accelerated.

### 3.3. Application of the RUSH System to Study the Cellular Trafficking of the K360del and K360A ABCG2 Mutant Variants

In order to examine the ER to plasma membrane trafficking of the ABCG2 variants, we applied a specific and efficient experimental method, the dynamic RUSH system [21], to the ABCG2 variants, as described in detail by Bartos and Homolya, 2021 [22]. In this method, the protein of interest is tagged with streptavidin-binding peptide (SBP) and GFP, whereas the ER-resident hook protein is tagged with streptavidin. When they are co-expressed, the protein of interest is retained in the ER, from where it can be released by the addition of biotin, and its trafficking can be tracked from the donor compartment to the target compartment. In this work, a GFP-SBP-ABCG2 fusion protein was co-expressed with an ER hook in HeLa cells. As documented in earlier studies, the N-terminally eGFP-fused ABCG2 was fully active and properly processed in mammalian cells [22,37].

The three variants of ABCG2 examined here were the WT, K360del and the K360A proteins (for details see the Methods section). The synchronized release from the ER initiated by biotin addition allowed us to monitor the cellular trafficking of the ABCG2 variants. To assess the kinetics of their cell surface appearance, an in situ immunostaining with a directly labeled 5D3 antibody was performed in non-permeabilized cells, and the colocalization of 5D3 labeling and the GFP fluorescence was determined. The time courses were fitted with a sigmoidal function and the kinetic parameters, including the initial values (CC_0_), the limits of the function (CC_inf_) and the time constants (k), were determined, as detailed in Bartos and Homolya [22].

**Figure 5:**
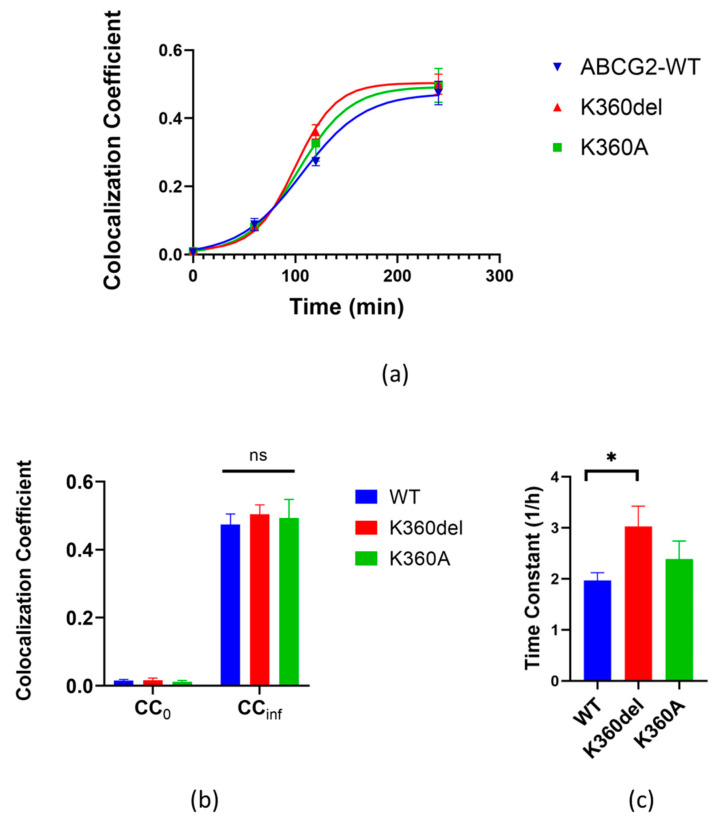
Kinetics of cell surface delivery of WT, K360del and K360A ABCG2 variants. (**a**) Colocalization coefficients of 5D3 antibody labeling and GFP fluorescence were determined using confocal microscopy images acquired at various time points. The kinetic curves were fitted with sigmoidal function. (**b**,**c**) Kinetic parameters of cell surface delivery were derived from the fitted kinetic curves: the initial values (CC_0_) and the limits of function (CC_inf_), as well as the time constants (1/h). Data from three independent experiments for all variants. For statistical analyses, Student’s *t* test was used. Differences were considered significant when *p* < 0.05, marked with a star, ns: non-significant.

As shown in Figure 5, the appearance of the K360del variant in the plasma membrane was significantly faster than that of the wild-type ABCG2 (measurements at 2 h), while after 4 h there was no significant difference in the final levels of the variants in the plasma membrane. These data indicate faster cellular trafficking from the ER-released K360del ABCG2 variant compared to the wild-type protein.

### 3.4. Molecular Dynamics Simulation of the K360del Intracellular ABCG2 Loop (a.a. 354-367) Variant

**Figure 6:**
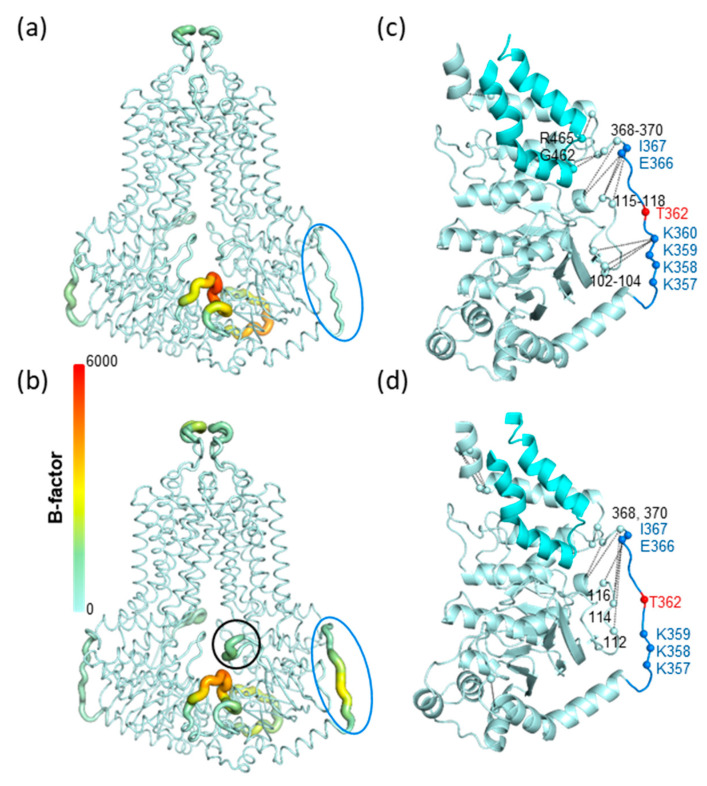
K360 mutations alter residue fluctuations and contacts in molecular dynamics simulations. (**a**,**b**). B-factors calculated from simulations are displayed in the context of WT and K360del structures. Thicker representation with warmer colors indicates higher dynamics. Blue circle: region of a.a. between 354 and 367; black circle: the gating helix of the regulation insertion. (**c**,**d**). Residue contacts characteristic for WT and K360del constructs (|difference| > 0.25) are indicated with black lines in the corresponding structures. Contacts were averaged along simulations and protomers, thus are indicated only on a single NBD-linker part of the structure. Pale cyan: NBD; cyan: coupling helix region (a.a. 441–483).

Microsecond long molecular dynamics simulations (3 × 1 μs for each construct) were performed with wild type, K360del and K360A ABCG2 embedded in a mixed membrane bilayer. The loop (a.a. 354-367), located after the second helix of the linker region and connecting the linker and the connecting helix before TM1, on average (3 simulations and 2 protomers), exhibited higher dynamics in the K360del ABCG2 variant as compared to the WT protein (Figure 6a,b). In order to investigate the source of this difference between wild type and K360del ABCG2, residue interactions from the MD trajectories were extracted (Figure S3). We highlighted those contact frequencies in the structures which exhibited a value larger than 0.25 when compared to the other construct (Figure 3 and Figure S3). 

The residues between 354 and 370 exhibited altered contacts in the K360del when compared to WT. The loop contacts characteristic for WT were decreased in the K360del variant (Figure 6c,d and Figure S3). Interestingly, K359 did not take over the interactions of K360, with residues L102, Ser103 and Gly104 residing in the S4 of NBD, a β-strand attached in an anti-parallel manner to S2 located after the regulatory insertion loop (Figure 6c,d). This indicates that, in spite of the mobility of the 554–567 loop, this segment is constrained in 3D without the possibility of wobbling. 

In summary, MD simulations suggested decreased NBD-linker interactions in the K360del variant with increased loop mobility. Moreover, the increased loop dynamics were allosterically communicated to the regulatory insertion in the K360 mutants, indicated by its increased dynamics (Figure 6a,b and Figure S3). Notably, this effect does not alter the ABCG2 function, as may be expected based on the homologous gating helices of ABC transporters in lower organisms [38,39,40].

## 4. Discussion

In this work, we have examined naturally occurring and artificially generated mutations in the unstructured cytoplasmic loop of the ABCG2 multidrug transporter. The aim of this study was to provide an analysis of the function and cellular processing of the mutant variants in a region which could not be resolved at the atomic level in any of the direct structural studies. In addition, this region has been previously suggested to be crucially important in ABCG2 [19]. 

In the case of the K360del, a naturally occurring mutation of ABCG2, previous studies indicated a basically unharmed expression and function, both in vivo and in vitro [17,18]. Here, we found a slightly increased level of plasma membrane expression in the K360del and K360A variants, and an accelerated trafficking between the ER and the plasma membrane for K360del, by applying the RUSH method. Both the K360del and the K360A variants had unchanged localization to the apical membrane regions. All these findings indicate that the K360del protein does not have an altered ribosomal translation and, while–probably exhibiting increased interactions with cellular proteins, travels faster to the plasma membrane. When exploring the potential effect of the K360del mutation on the cellular expression of the improperly processed Q141K variant, which is present in a large amount of the human population, we did not find a rescue of this variant (see Figure 2c for protein expression). 

Molecular dynamics studies suggest an increased mobility of the unstructured loop in the K360del variant. The increased dynamics of the loop may increase the availability of binding sites for protein–protein interactions. This may result in altered interactions with proteins playing a role in trafficking from the ER through the Golgi apparatus to the plasma membrane, as indicated by the RUSH experiments. The increased dynamics and accessibility for protein–protein interactions may also result in increased interactions with proteins at the plasma membrane (e.g., [35]), potentially contributing to increased steady state levels on the cell surface.

In this study, we have also examined in detail the function, processing and degradation of the K357A, K358A, K359A, K357-360A, as well as T362A and T362E, variants of the ABCG2 protein. We also explored the effects of the protein synthesis inhibitor cycloheximide on the cellular fate of the K360del and T362A variants and found no major differences between the plasma membrane retrieval of these variants and the wild-type protein. 

It has to be emphasized that we did not find any major effect from the T362A or T362E variants on the expression, trafficking or function of the ABCG2 protein. These results are in strong contradiction to an earlier report [19], considering an important and specific role of the PIM kinase-dependent phosphorylation of this residue, obtained by an N terminally HA-tagged ABCG2 protein. The direct threonine replacements in our studies using the untagged protein variants strongly question the regulatory role of such a phosphorylation. 

As a summary, the residues examined by us in the flexible loop region (a.a. 354-367) in the ABCG2 protein may slightly modify the expression, trafficking and plasma membrane expression levels, while having no significant effects on the function and apical membrane localization of this multidrug transporter. Importantly, our experiments reinforce the harmless and slightly beneficial effect of the naturally occurring K360del variant on ABCG2 expression. These results also exclude the frequently discussed potential effect of the specific phosphorylation of the T362 residue on ABCG2 function or localization. Our results may initiate further efforts for exploring the potential protein–protein or protein-lipid interactions of this flexible loop in ABCG2.

## Data Availability

All data are available upon request.

## Supplementary Materials

The following supporting information can be downloaded at: https://www.mdpi.com/article/10.3390/membranes13100822/s1. Figure S1: Hoechst33342 accumulation with or without the Ko143 inhibitor in HEK293 cells expressing the ABCG2 variants; Figure S2: Root mean square deviation (RMSD) of conformations from the initial structure indicated stable simulations; Figure S3: Characteristic pairwise residue contacts between the a.a. 330–390 and the rest of the protein.
