# Supplementary material for: Expression, Function and Trafficking of the Human ABCG2 Multidrug Transporter Containing Mutations in an Unstructured Cytoplasmic Loop
Source: Membranes (Basel). 2023 Oct 4;13(10):822. doi: 10.3390/membranes13100822 (PMC10608301; doi:10.3390/membranes13100822)
Supplement: Supplementary file 1 [file membranes-13-00822-s001.zip › membranes-2623053-supplementary.pdf]

SUPPLEMENTARY MATERIAL

Expression, function and trafficking of the human ABCG2 multidrug transporter containing mutations in an unstructured cytoplasmic loop

Orsolya Mózner, Boglárka Zámbó, Zsuzsa Bartos, Anna Gergely, Kata Sára Szabó, Bálint Jezsó, Ágnes Telbisz, György Várady, László Homolya, Tamás Hegedűs and Balázs Sarkadi

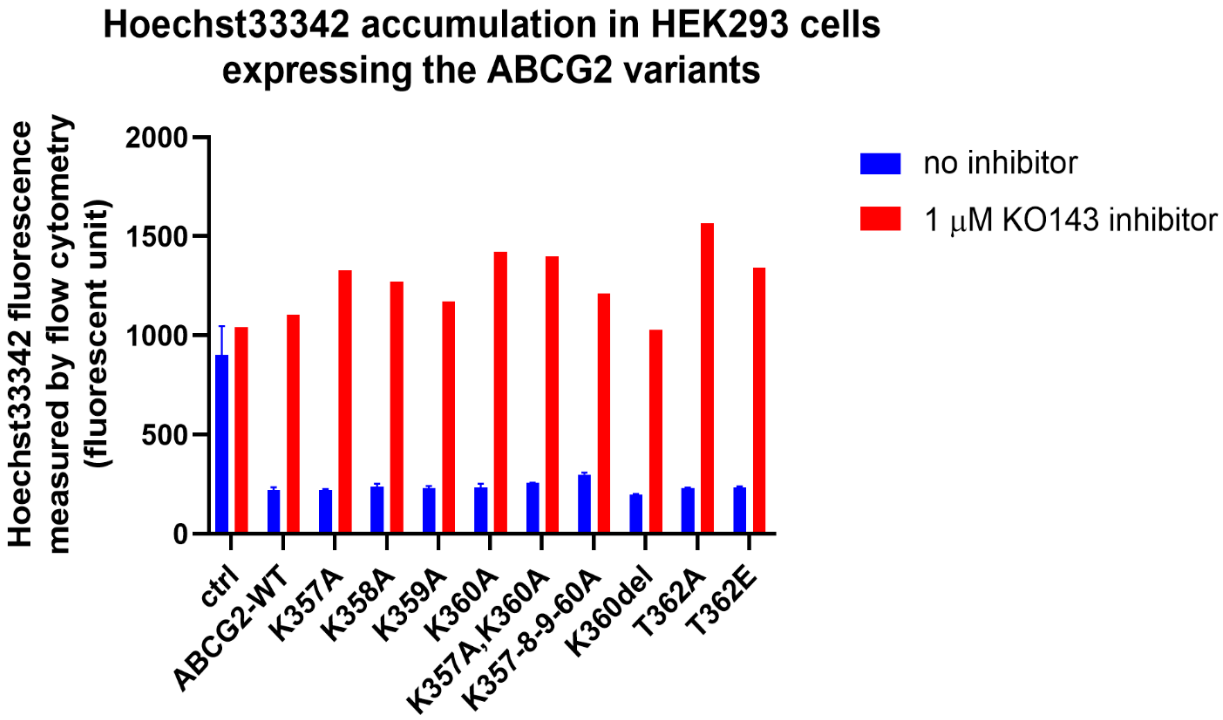

Supplementary Figure S1. Hoechst33344 dye accumulation in HEK293 cells expressing the ABCG2 variants.

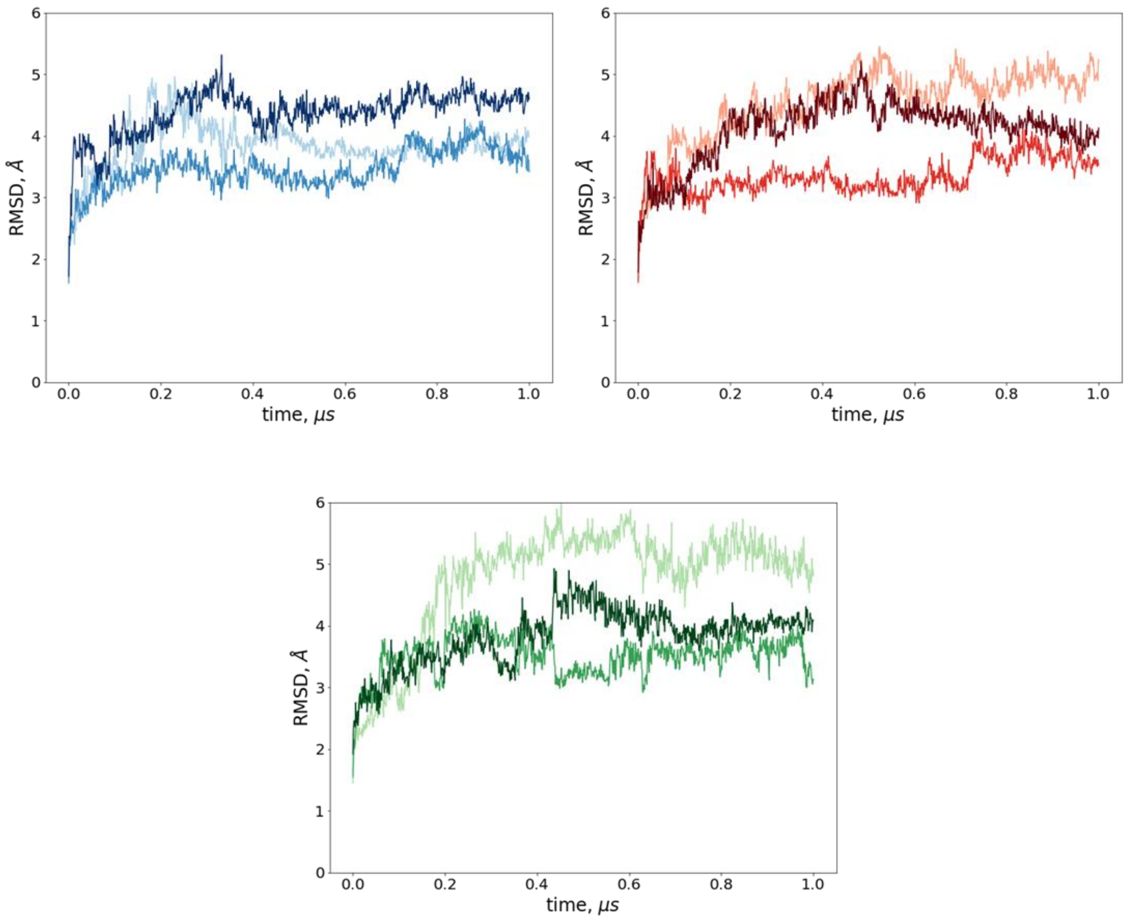

Supplementary Figure S2. Root mean square deviation (RMSD) of conformations from the initial structure. The values indicate stable systems of the size of ABCG2, with a mobile loop between NBD and TMD, which contribute to the majority of differences from the starting conformation (see Figure 6).

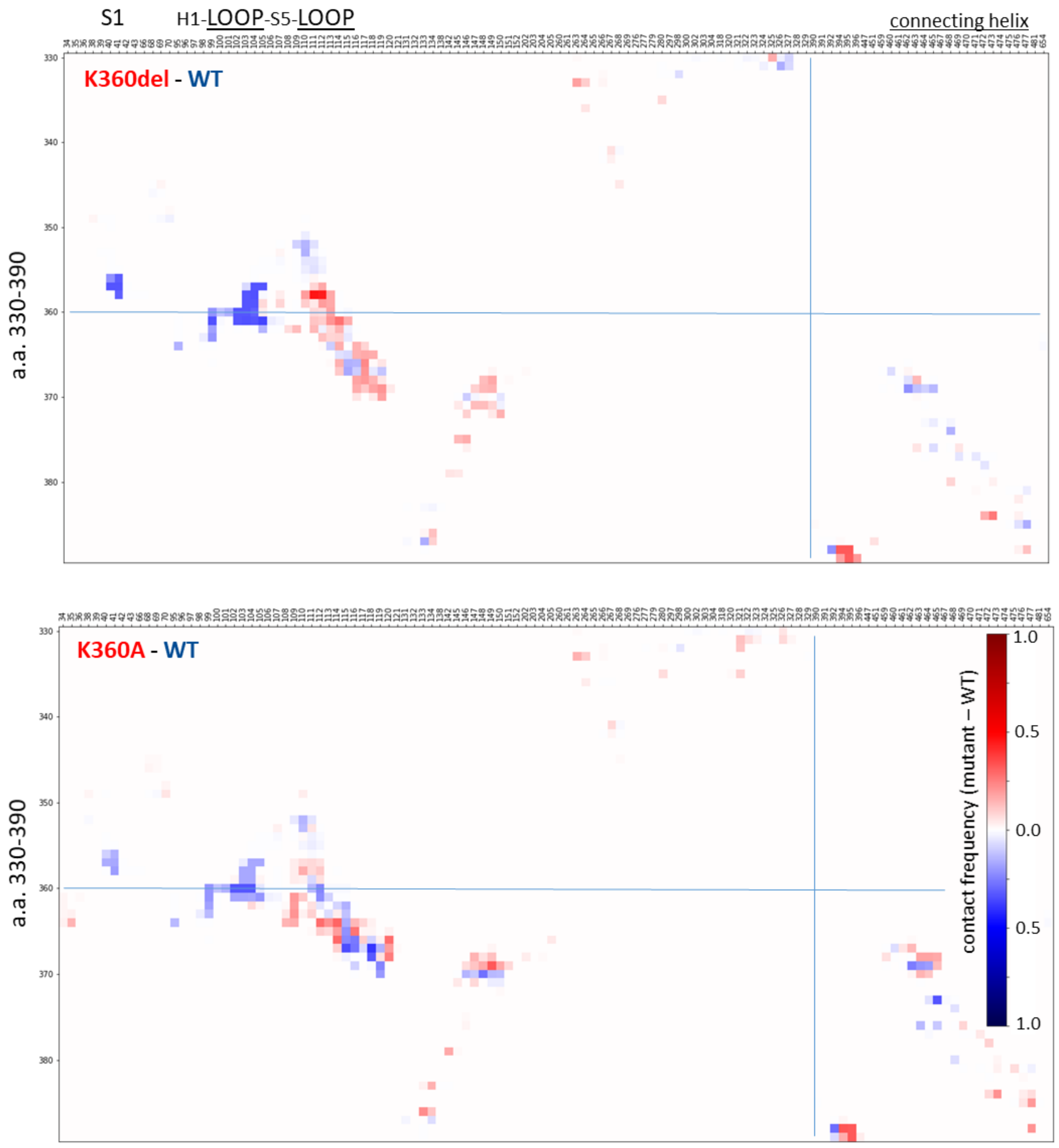

Supplementary Figure S3. Characteristic pairwise residue contacts between the a.a. 330-390 and the rest of the protein. Contact maps were calculated (between Cα with 7.5 Å cutoff) for each construct. To display the most characteristic interactions, the WT map was subtracted from the mutant and only those residues are shown which contact the loop. The contacts present with higher frequencies in the WT are blue and the contacts present more in the K360del and K360A are red. The horizontal and blue lines labels indicate the position 360 (or 361 in K360del) and the location of a.a. 330-390, respectively. This latter region is not shown in the X axis to hide self-interactions for clarity. Both mutations decrease the interactions of the disordered loop (a.a. 354-370) with the first β-strand of NBD1 (S1) and with the loop between the H1 helix containing Walker A and β-strand S5. The interactions of this 354-370 region with the loop next to S5 are characteristic more for K360del than for the K360A or WT.
